# When can AI psychotherapy be considered comparable to human psychotherapy? Exploring the criteria

**DOI:** 10.3389/fpsyt.2025.1674104

**Published:** 2025-09-24

**Authors:** Nick Kabrel

**Affiliations:** ^1^ Department of Psychology, University of Zurich, Zurich, Switzerland; ^2^ Digital Society Initiative, University of Zurich, Zurich, Switzerland

**Keywords:** psychotherapy, chatbot, AI, RCT, ChatGPT

## Main text

An increasing number of randomized controlled trials (RCTs) demonstrate the efficacy of psychotherapy delivered by artificial intelligence (AI)-based chatbots (reviewed in (1–3)). For instance, a recent first-of-its-kind RCT of a generative AI chatbot found a large effect size for depression reduction at 8 weeks (Cohen’s d ≈ 0.8) (4). Picked up by industry stakeholders, media outlets, and even some researchers, this has led to the claims that AI psychotherapy might already be comparable to human psychotherapy. Anticipating more studies showing similar results, a crucial question emerges: Do comparable effect sizes imply equivalence between AI chatbot therapy and human psychotherapy, such that they can be considered interchangeable treatment options?

This perspective argues that while symptom-level outcome comparability is an important milestone, it alone does not establish equivalence. True equivalence requires meeting a broader and more nuanced set of empirical and theoretical criteria that capture the complexity of psychotherapy as a clinical practice. Currently, these criteria are largely absent from the literature, with only a few papers so far formally discussing the comparability of chatbot- and human-based therapies (see, e.g (5, 6)). In the attempt to contribute to this discussion, we propose an exploratory set of criteria to guide rigorous evaluation and inform clinical, ethical, and policy decisions.

For example, one long-standing limitation of Digital Mental Health (DMH) interventions is the high attrition rates (7–9). RCTs often show efficacy in controlled settings with motivated participants, whereas real-world environments are more varied and less structured. Traditional psychotherapy benefits from a unique form of human accountability that sustains engagement despite fluctuations in motivation or external stressors. The absence of at least minimal human-to-human contact in chatbot-based interventions may limit sustained use and thereby reduce long-term effectiveness (7). Claims of comparability must therefore be supported by studies demonstrating sustainable real-world adherence and engagement.

In addition, the implementation of DMH tools within established healthcare systems remains a significant challenge (8). Interventions tested in isolation (e.g (4)) may fail when integrated into real-world workflows. For chatbot-based therapy to be considered comparable, it must demonstrate not only stand-alone efficacy but also effective incorporation into healthcare settings, including primary care, mental health clinics, and stepped-care models (9). Previous DMH tools, despite showing efficacy, often failed in practice due to misalignment with patient needs or clinician workflows (e.g., see (9, 10)). Therefore, feasibility studies are essential to assess whether chatbot therapy is viable and compatible with daily clinical workflows.

The APA’s recent ethical guidance for AI in psychological services contributes to solving this issue by emphasizing that AI tools must be designed with user-centered features, such as culturally responsive interfaces and personalized feedback, to enhance engagement in diverse real-world populations (11). It also emphasizes the human-in-the-loop approach that might help to mitigate engagement concerns. For example, studies show that at least minimal involvement of a human in the digital intervention (e.g., in the form of coaching) might help retain people (10).

Furthermore, current RCTs evaluating chatbot interventions primarily report short-term outcomes, often limited to 6–8 weeks (e.g (4, 12, 13)). In contrast, psychotherapy research emphasizes long-term change, including relapse prevention and maintenance of gains. Without longitudinal follow-up data, ideally extending at least 6–12 months, claims about the therapeutic potential of chatbots remain provisional. Future studies should assess time to relapse, symptom recurrence, and long-term functioning to determine if chatbot therapy fosters enduring change.

Another important issue is that therapeutic change often arises from the quality and dynamics of the therapeutic relationship (14). While chatbot interactions can simulate empathy and responsiveness, it is unclear whether they can replicate deeper interpersonal processes, such as the formation and resolution of alliance ruptures. Decades of psychotherapy research show that effective therapy is not only about empathy and affirmation but about navigating tension, misalignment, negative emotions toward the therapist, and repair, all of which contribute to psychological growth (15, 16). It remains an open question whether AI systems can support these fundamentally human relational processes. This is especially relevant in treating personality disorders or relational trauma, where change depends on corrective relational experiences (17, 18).

As an expansion of the point made above, it is well-known that human psychotherapy can lead to deep psychological transformations beyond mere symptom reduction (19). While symptom reduction is a valuable metric for RCTs, it does not fully capture lasting and meaningful psychological transformation. Shedler (20) lists criteria for mental health beyond symptom reduction, including, for example, (a) being able to form close and lasting friendships characterized by mutual support and sharing of experiences, (b) coming to terms with painful experiences from the past and finding meaning in and grown from such experiences, and (c) being capable of sustaining a meaningful love relationship characterized by genuine intimacy and caring. It is unclear whether chatbot therapy can facilitate such a transformation beyond mere symptom reduction. Note that although these outcomes are not achieved in every case with human therapy, it has at least demonstrated the potential to produce them.

An additional challenge is that most studies of chatbot interventions focus on mild-to-moderate depression and anxiety (1–4). These samples often exclude individuals with severe, comorbid, or high-risk conditions. Human psychotherapists are trained, and healthcare systems are prepared to navigate diagnostic ambiguity, emotional complexity, and clinical risk. In contrast, chatbot systems lack grounding in contextual understanding and may respond inappropriately to delusional thinking, self-harm ideation, or trauma disclosures (e.g., affirm and reassure these tendencies due to inherent sychophancy). To be deemed comparable in a general sense, chatbot therapy must either demonstrate efficacy across a broader spectrum of psychopathology or be explicitly positioned as symptom-managing tools for circumscribed conditions (e.g., mild depressive symptoms).

Relevant to this issue is the entry point into care. People access therapy under very different circumstances. Some enter treatment during an acute crisis (e.g., suicidality, abrupt relational changes), where risk management is essential. Others begin therapy when they are relatively stable but motivated for deep, long-term change. A third group may only seek psychoeducation, information, or short-term skills training. Human psychotherapy is designed to flexibly accommodate this diversity of goals, whereas chatbot therapy may only be suited for a narrower range (e.g., psychoeducation or structured short-term interventions). Therefore, claims of comparability must be carefully considered in the context: for whom and for what aims chatbot interventions are intended. Equivalence cannot be assumed across all entry points and therapeutic goals. Rather, chatbot-based therapy should be explicitly positioned within a limited scope of use cases and evaluated accordingly.

Finally, despite constant technological developments, ethical and risk concerns still remain a big challenge for chatbot-based mental health interventions (21). Unlike human therapists, who undergo licensing processes that ensure extensive training, supervision, and accountability, chatbots cannot be considered licensed practitioners. While they may be trained on therapy manuals and overseen by clinicians or engineers, this is not equivalent to professional preparation or responsibility. Moreover, ethical risks extend beyond licensing. Chatbots may fail to adequately detect crisis situations (e.g., suicidality or psychotic episodes), may inadvertently reinforce harmful cognitions, or may not provide appropriate escalation pathways to human care. Issues of data privacy, bias and cultural adjustments, and informed consent further complicate deployment.

In addition to the conceptual and clinical concerns outlined above, in what follows, we address methodological and design considerations that must be met in future RCTs for claims of comparability between AI-based and human-delivered psychotherapy to be valid.

First, true comparability requires head-to-head studies between chatbot and human-delivered psychotherapy using robust methodologies. Ideally, they should use non-inferiority designs, strong randomization, and control for expectancy/placebo effects.

Second, in the recent study by Heinz et al. (4)—a state-of-the-art RCT that dictates the chatbot therapy narrative—no blinding procedures were implemented. This omission presents a significant methodological limitation, particularly given the advanced language capabilities of current AI chatbots, which may lead participants to form heightened expectations. To strengthen causal inference, future studies should incorporate carefully designed control conditions. A control chatbot could mimic conversation but omit therapeutic mechanisms, acting as a neutral digital companion. Without this level of experimental control, reported effect sizes may overestimate the true therapeutic impact of AI interventions.

Third, current trials often recruit participants through mainstream social media (4), digital platforms, or mental health apps, resulting in samples that do not reflect typical psychotherapy populations. A recent systematic review suggests that online samples significantly differ from traditional populations that use psychotherapy services in terms of demographics, severity, and treatment history (22). Current chatbot RCTs primarily recruit individuals who are already motivated and willing to engage, often in a non-crisis state. This limits generalizability, as many real-world patients initiate therapy in crisis or with ambivalent readiness to change. For conclusions to generalize, studies should recruit from naturalistic clinical settings and ensure representativeness across age, socioeconomic status, cultural background, and clinical presentation.

In conclusion, the criteria proposed here offer a preliminary framework for evaluating whether an AI chatbot-delivered psychotherapy can be considered comparable to human psychotherapy (see also **Table 1** for summarization). At present, claims of equivalence and even comparability are premature and risk misleading both policymakers and the public. In the absence of rigorous theoretical grounding and empirical validation, there is a danger of overstating the readiness of chatbot-based interventions as substitutes for human care. We argue that unless clearly defined criteria are met, chatbot psychotherapy should not be marketed or presented as legitimate psychotherapy, nor as equivalent or comparable to human-delivered care. We hope to inspire further research and debate on the appropriate criteria for establishing therapeutic comparability in mental health care, and to encourage researchers to propose additional or alternative criteria to help the field progress.

**Table 1:** Critical gaps in current chatbot interventions that must be addressed before claims of equivalence can be substantiated.

Component	Description	Human-delivered therapy	AI-Delivered therapy
Implementation challenges			
*Engagement*	Ability to establish and maintain active user involvement over time	Yes	Limited evidence
*Implementation*	Real-world feasibility and fidelity of therapeutic delivery	Yes	Limited evidence
*Flexibility and context*	Capacity to address diverse entry contexts (crisis, long-term change, short-term/psychoeducation)	Yes	Not sufficiently addressed
Outcomes of psychotherapy			
*Long-term outcomes*	Evidence for sustained benefits years after the intervention	Yes	No
*Relational change*	Capacity to foster deep relational change via working through therapeutic relationships	Yes	No
*Changes beyond symptom reduction*	Development of enduring relational capacities, emotional depth, and integration of past experiences	Yes	No
Ethical and risk concerns			
*Severe psychopathology management*	Safe and effective treatment of complex, high-risk cases	Yes	No
*Ethical safeguards*	Demonstrated capacity to ensure privacy, provide clear escalation to human care, and establish accountability structures equivalent to licensed practice.	Yes	No
Methodological issues			
*Blind studies*	Use of proper blinding to control expectancy and placebo effects	Yes	No
*Naturalistic recruitment*	Inclusion of participants reflecting typical clinical populations	Yes	No
*Direct comparability studies*	Trials that test AI vs. human therapy under equivalent conditions	No	No
